# Parent-Offspring Correlations in Pedometer-Assessed Physical Activity

**DOI:** 10.1371/journal.pone.0029195

**Published:** 2011-12-28

**Authors:** David Jacobi, Agnès Caille, Jean-Michel Borys, Agnès Lommez, Charles Couet, Marie-Aline Charles, Jean-Michel Oppert, FLVS Study Group

**Affiliations:** 1 Service de Médecine Interne-Nutrition, Centre Hospitalier Régional Universitaire (CHRU) de Tours, Tours, France; 2 Institut National de la Santé et de la Recherche Médicale (INSERM) U 921, Université François Rabelais de Tours, Tours, France; 3 Institut National de la Santé et de la Recherche Médicale (INSERM) Centre d'Investigation Clinique (CIC)-202, Université François Rabelais de Tours, Tours, France; 4 Fleurbaix-Laventie Association, Laventie, France; 5 Institut National de la Santé et de la Recherche Médicale (INSERM) U 1018, Centre de Recherche en Epidémiologie et Santé des Populations (CESP), Team Lifelong Epidemiology of Obesity, Diabetes and Renal Disease, Villejuif, France; 6 Université Paris–Sud XI, Kremlin-Bicêtre, France; 7 Service de Nutrition, Hôpital Pitié-Salpêtrière (Assistance Publique des Hôpitaux de Paris - AP-HP), Paris, France; 8 Centre de Recherche en Nutrition Humaine Ile-de-France (CRNH-IdF), Université Pierre et Marie Curie-Paris 6, Paris, France; University of Massachusetts Medical School, United States of America

## Abstract

**Background:**

Physical activity is a major component of a healthy lifestyle in youth and adults. To identify determinants of this complex behavior is an important research objective in the process of designing interventions to promote physical activity at population level. In addition to individual determinants, there is evidence documenting familial influences on physical activity. However, the few studies that have addressed this issue with objective measures did not provide data on parent-offspring physical activity relationships throughout childhood and adolescence. The purpose of this study was to assess familial correlations in pedometer-assessed physical activity.

**Methods:**

We measured ambulatory activity in 286 French nuclear families (283 mothers, 237 fathers, and 631 children aged 8–18 years) by pedometer recordings (Yamax Digiwalker DW 450) over a week. Correlations were computed with their 95% confidence intervals (CI) for spouse pairs, siblings, mother-offspring, and father-offspring. Data were expressed as steps per day and computed both for the full recording period and separately for weekdays and weekends.

**Results:**

The correlations were the highest between siblings (r = 0.28, 95%CI: 0.17–0.38). Parent–offspring correlations were significant in mothers (r = 0.21, 95%CI: 0.12–0.30), especially between mothers and daughters (r = 0.24, 95%CI: 0.12–0.36 vs. r = 0.18, 95%CI: 0.05–0.31 for sons), but were almost nonexistent in fathers. Correlations were generally higher on weekend days compared to weekdays. Mother-offspring correlations did not decrease with increasing age of children (r = 0.17, 95%CI: 0.00–0.34 in 8–11-year-olds, r = 0.20, 95%CI: 0.07–0.33 in 12–15-year-olds, and r = 0.25, 95%CI: 0.07–0.39 in ≥16-year-olds). Finally, between-spouse correlations were significant only during weekend days (r = 0.14, 95%CI: 0.01–0.27).

**Conclusion:**

Ambulatory activity correlated within families, with a possible mother effect. Mother-offspring correlations remained significant through the transition from childhood to adolescence. Further studies are required to better understand the respective influences of shared activities, parental modeling and support as well as genetic factors on the familial aggregation of physical activity.

## Introduction

Physical activity is now recognized as a major component of a healthy lifestyle in children and adolescents as well as in adults. Physical activity in youth provides many physiological and psychological health benefits and may also continue into adulthood [1], [2], [3]. There has been a long-standing research interest about familial correlates of children and adolescents' physical activity levels that could justify family based interventions [4], [5], [6]. However, findings from studies on the relationship between physical activity levels of parents and those of their children have been mixed.

The current knowledge on how physical activity correlates between parents and offspring mainly relies on assessments by recall data whether provided by the parents themselves (self-reports and reports for the child) or by the children (perceived' parental physical activity levels) [4], [5], [6]. Objective measures of physical activity, as provided by movement counters, would be expected to give greater accuracy in quantifying these relationships. Among these instruments, accelerometers are able to assess physical activity duration, intensity, and frequency and pedometers (or step-counters) provide an inexpensive overall measure of physical activity [7]. However, up to now, few studies have examined resemblance of physical activity between parents and offspring using such methods.

Two early studies performed at the beginning of the 90's used accelerometers but in small and selected samples of families and found some degree of parent-offspring aggregation of physical activity [8], [9]. A more recent body of research shows the current interest for the use of objective methodologies. Using accelerometry, Jago et al. did not observe significant correlations between parent and child physical activity [10] whereas other reports found that parents' physical activity levels predicted those of children [11], [12]. Ambulatory activity (walking, running) represents the most commonly and easily performed type of physical activity. Walking, as a typical moderate-intensity physical activity, forms the basis of current physical activity recommendations [2]. Although, accelerometers provide detailed data for physical activity (including intensity), pedometers more specifically assess ambulatory activity which is then quantified in steps per day [7]. However, it is not known whether parent-offspring physical activity correlations are evident when pedometers are utilized. The only study that provided concomitant pedometer data for both parents and offspring did not report on the familial correlation [13].

Of note, the latter studies were limited to a short age range of children as recruitment relied on school grades as opposed to a nuclear family-based recruitment that would have included all siblings within the family [8], [9], [10], [11], [12]. As a result, there is also a lack of data about the evolution of ambulatory activity in the offspring from childhood to adolescence and its relationship with that of their parents. Studies addressing this issue seem important in light of the established finding that physical activity in youth decline with age, especially throughout adolescence [14], [15].

Taking advantage of pedometer recordings collected in nuclear families in a French population sample, we examined familial aggregation in objectively measured ambulatory activity.

## Methods

### Objectives

The primary objective of this study was to examine familial aggregation in pedometer-assessed ambulatory activity by assessing parent-offspring correlations under daily life conditions. Another objective was to investigate whether correlations would change with increasing age of the offspring.

### Participants

Nuclear families, defined as family groups consisting of a father and/or mother and their children who share living quarters, were drawn from the Fleurbaix-Laventie Ville-Santé II (FLVS II) study, a prospective study (1999–2001) aimed at identifying determinants of adiposity and its changes over time in families living in two small towns in Northern France, Fleurbaix and Laventie [16], [17]. The target population included approximately 1,500 adults within 393 families, of which 294 (75%) agreed to participate in the study after a call in the local medias. Children and adolescents were asked to participate in the study along with their parents. The choice of the cut-off for age (8 years) was related to difficulties of younger children in understanding the requirements of the study for accurate participation.

### Description of procedures

Research assistants met the participants at their home. Each participant was provided with a Yamax Digiwalker DW 450 (Yamasa Corporation, Tokyo, Japan) pedometer and with a diary so that they could record every evening the number of steps walked each day. Research assistants made the appointments, showed the participants how to operate the pedometer, and gave oral instructions to parents on how to record their and their children's number of steps taken each day in the diary. The pedometer was worn on the belt during waking hours for 7 consecutive days. Advice was given to both the parents and children to follow their usual physical activity routine. Self-declared past-year leisure-time physical activity (LTPA) was assessed in parents using the Modifiable Activity Questionnaire (MAQ) administered at the initial visit by trained interviewers. Body weight was measured in light clothes to the nearest 0.1 kg using a bipedal bio-electrical impedance device (Tanita TBF 310 model; Tanita, Courbevoie, France) and standing height without shoes to the nearest centimeter using a portable stadiometer. Data from the initial visit were used for the present paper.

### Ethics

The study protocol was approved by the regional Ethics Committee (Comité Consultatif de Protection des Personnes se prêtant à des Recherches Biomédicales (CPPRB) de Lille, Hôpital Huriez, 59037 Lille, France). All parents gave their written informed consent.

### Statistical methods

The data are presented as means ± standard deviation and as median and interquartile range for normally and non-normally distributed data, respectively. Wilcoxon rank tests were used to compare non-normally distributed number of steps between different categories of individuals. Spearman correlation coefficients were compared with Z test on Z transformed values.

#### Analysis of familial associations

Four different variables were created for pedometer data: mean number of daily steps over 1 week (≥4 days of pedometry recording required for the correlation analysis in accordance with previous recommendations in youth [18] and adults [19]), mean number of daily steps during weekdays (≥2 days of pedometry recording required for the correlation analysis), mean number of daily steps during weekend days (≥1 day of pedometry recording required for the correlation analysis), and number of steps taken on Wednesdays (a day in the week when children are off school in France and adolescents attend school for half a day). In order to estimate the correlations, each of these variables was standardized according to age and gender with a *z*-score [20]. The *z*-score is defined by *Z* = (*x* − *Mi*)/*Si* with *Mi* and *Si* corresponding to the mean and standard deviation specific to gender and age category of the considered individual. For the offspring, the age categories were defined as follows: 8–11, 12–15, and ≥16-year-old. For the analysis, the participants were grouped as parents, mothers, fathers, offspring, sons, and daughters.

The familial correlations were estimated by intraclass correlation coefficients (ICC), which quantify the degree of resemblance between any two members of the same category of individuals in a family and by interclass correlation coefficient which quantify the degree of resemblance between any two members from different classes of individuals in a family. Higher scores imply a stronger familial resemblance. Between-spouse correlations were computed with Pearson product-moment correlation. Sibling correlations were computed with intraclass correlation coefficient (one way analysis of variance) and 95% confidence interval (CI) according to Searle's method [21]. For parent (mother or father)-offspring correlations, interclass correlation coefficients were estimated with the pairwise estimator described by Rosner et al. [22]. The 95%CI was estimated with a method based on a modification of a Fisher transformation. For all correlation coefficients, negative values were truncated to 0. All analyses were carried out using SAS software (Version 9.1 of SAS system for Windows; SAS Institute Inc., Cary, NC) or R Project for Statistical Computing v2.8.1. [23].

## Results

From the initial 294 families (1,168 individuals), eight families were excluded because no pedometer data were available either for the parents or their offspring, thus the final number of families was 286 (1,151 individuals). The number of children per family was: one in 53 families (18.5%), two in 142 families (49.7%), three in 75 families (26.2%), four in 11 families (3.9%), and five in 5 families (1.8%). Most families (81.2%) had two parents.

### Descriptive characteristics

The characteristics of the study population are shown in Table 1, by gender and by age groups in children. Table 2 shows the summary data for pedometer recordings. In the offspring, boys took significantly more steps than girls (median 9453 (interquartiles: 7149–11819) vs. 7770 (6168–9567), p<0.0001) and there was a consistent decrease in number of steps taken with increasing age (Spearman r = −0.25, 95% CI: −0.33–−0.17). This decrease was more pronounced among boys when compared to girls (boys Spearman r = −0.29, 95% CI: −0.39–−0.18 and girls r = −0.26, 95% CI: −0.36–−0.14, Z test, p<.0001). Fathers walked 9% more steps than mothers during both weekdays and weekend days (p = 0.07 and p = 0.10, respectively).

**Table 1:** Characteristics of the subjects (Fleurbaix-Laventie Ville-Santé II study).

	Girls			Boys			Mothers	Fathers
N	79	120	114	72	141	104	283	237
Age (years)	8–11	12–15	≥16	8–11	12–15	≥16	42.4±4.6	44.2±5.1
Weight (kg)	35±8	50±10	58±9	34±8	52±12	69±12	66±13	81±15
Height (cm)	141±9	161±7	166±6	143±10	164±11	178±6	163±6	176±6
BMI (kg m^−2^)	17.3±2.8	19.2±3.0	21.1±3.0	16.4±2.2	19.0±3.1	21.8±3.8	24.8±4.9	25.9±4.2
Leisure time (h·week^−1^)							2.3 (1.1–3.8)	3.5 (1.8–5.8)
Walking (h·week^−1^)							0.46 (0–1.38)	0.12 (0–0.81)
Work (h·week^−1^)							39.9 (31.9–39.9)	42.1 (39.9–49.9)
Employed, n (%)							199 (71.8)	222 (95.3)

Values are expressed as mean ± standard deviation, or median and interquartiles. Data for leisure time and walking are from the Modifiable Activity Questionnaire.

**Table 2:** Pedometer counts (steps per day) (Fleurbaix-Laventie Ville-Santé II study).

	Girls			Boys			Mothers	Fathers
	8–11 y	12–15 y	≥16 y	8–11 y	12–15 y	≥16 y		
All week	8432 (7231–9775)	8475 (6532–9819)	6688 (5038–8440)	11030 (8412–14028)	9508 (7606–11350)	7942 (6473–10519)	7273 (5776–9231)	8091 (5993–9899)
Weekdays	8631 (7179–10153)	8304 (6604–10081)	7028 (5061–9071)	10900 (8454–14427)	9416 (7347–11476)	7965 (6560–10734)	7403 (5629–9504)	8116 (5704–10143)
Weekend days	7654 (5372–10552)	8045 (5793–10597)	5770 (4460–8218)	10888 (7732–15531)	9239 (6561–12377)	7283 (4659–9436)	6918 (4904–9179)	7474 (5166–9906)
Wednesdays	8166 (6238–10511)	7277 (5195–11229)	6626 (4609–9670)	10309 (5807–12830)	8784 (5867–12319)	7740 (4606–11041)	7254 (5200–9852)	8018 (5229–10894)

Values are daily medians and interquartiles.

### Intrafamily correlations

Table 3 shows the correlations in number of steps per day within the family. Correlations were generally the highest between siblings. Between-spouse and parent-offspring correlations were higher during weekend days. Most of the significant parent-offspring correlations were found between mothers and offspring, especially in the mother-daughter pairings. There was a mother effect rather than a same-sex parent effect, and most fathers-offspring correlations were not significant. These correlations generally increased as the offspring age class increased and were higher for daughters when compared to sons.

**Table 3:** Familial correlations for pedometer-assessed physical activity (Fleurbaix-Laventie Ville-Santé II study).

	Between-spouse	Siblings	Mother-offspring	Father-offspring
Maximal number of subjects in the analysis	438	584	834	718
Maximal number of clusters	219	274	265	225
All week	0.05 (0.00–0.18)	0.28 (0.17–0.38)	0.21 (0.12–0.30)	0.01 (0.00–0.12)
Weekdays	0.02 (0.00–0.15)	0.25 (0.14–0.35)	0.15 (0.06–0.24)	0.00 (0.00–0.09)
Weekend days	0.14 (0.01–0.27)	0.24 (0.14–0.35)	0.25 (0.15–0.34)	0.05 (0.00–0.15)
Wednesdays	0.06 (0.00–0.20)	0.31 (0.20–0.42)	0.15 (0.04–0.24)	0.02 (0.00–0.13)

Data are correlation coefficients (95%CI) of number of steps standardized for sex and age.

Maximal number of clusters because for each coefficient computation data could be missing.

Table 4 shows the stratified correlations in number of steps per day between mothers and their offspring. Correlations were higher in mothers who were employed and who declared leisure time physical activity above the median of the population. Fathers-offspring correlations were only significant during weekend days for children ≥16-year-old (ICC = 0.19, 95%CI: 0.02–0.35).

**Table 4:** Mother-offspring correlation for pedometer-assessed physical activity (Fleurbaix-Laventie Ville-Santé II study).

	Depending on mother's characteristics				Depending on child's age and sex				
	Employed		Leisure time physical activity		8–11 y	12–15 y	≥16 y	Daughters	Sons
	Yes	No	≥2.32 h·week^−1^	<2.32 h·week^−1^					
n	597	237	412	422	255	431	335	479	481
Clusters (maximal)	191	75	129	136	115	194	143	192	199
All week	0.24 (0.13–0.35)	0.11 (0.00–0.29)	0.24 (0.10–0.36)	0.18 (0.04–0.31)	0.17 (0.00–0.34)	0.20 (0.07–0.33)	0.25 (0.11–0.39)	0.24 (0.12–0.36)	0.18 (0.05–0.31)
Weekdays	0.17 (0.06–0.28)	0.10 (0–0.28)	0.18 (0.05–0.31)	0.11 (0–0.24)	0.08 (0–0.25)	0.18 (0.05–0.31)	0.17 (0.02–0.31)	0.20 (0.07–0.31)	0.11 (0–0.24)
Weekend	0.29 (0.19–0.39)	0.14 (0–0.31)	0.27 (0.14–0.39)	0.19 (0.06–0.32)	0.21 (0.03–0.36)	0.17 (0.04–0.30)	0.35 (0.21–0.48)	0.23 (0.10–0.35)	0.26 (0.14–0.38)
Wednesday	0.19 (0.07–0.31)	0 (0–0.19)	0.21 (0.07–0.35)	0.06 (0–0.20)	0.13 (0–0.32)	0.13 (0–0.26)	0.19 (0.03–0.34)	0.18 (0.05–0.31)	0.11 (0–0.24)

Data are interclass correlation coefficients (95%CI) of number of steps standardized for sex and age. Data for physical activity during leisure time are from the Modifiable Activity Questionnaire. The total number of clusters can differ from maximal number of clusters (283) because of missing data.

## Discussion

We conducted a study of familial aggregation of ambulatory physical activity in a free-living population. This study provides the largest population dataset among the studies that used objective measures of physical activity with these aim and setting. Another original feature is the involvement of nuclear families including all the siblings. The present study analyzing pedometer recordings indicate the presence of familial aggregation of ambulatory activity. Parent-offspring correlations were only significant in mothers, and stronger between mothers and daughters. Higher correlations were found during weekend days. Finally, mother-offspring correlations remained of the same magnitude through the transition from childhood to adolescence.

The finding of a similarity between physical activity of parents and their children has several possible explanations. These include the parents acting as role models, sharing of activities, and support by active parents [24]. In addition, several types of data support a role of genetic factors not only for the determination of traits related to training response but also for usual physical activity levels, and emerging evidence suggests potential genomic locations for these genetic influences [25]. However, the finding of correlations between parents and offspring that were only significant for mothers was less expected. In a review of environmental correlates of physical activity in youth, Ferreira et al. concluded that despite most studies failing to find any association, fathers appear to be more important role models compared to mothers, especially in childhood [5]. Fuemmeler et al., using accelerometer-based measures of moderate to vigorous physical activity, found moderate to high correlations between mothers and daughters (r = 0.67, p<0.01) and between fathers and sons (r = 0.43, p<0.05) whereas correlations were non-significant in pairs of opposite genders [12]. In a large sample of 2,375 nuclear families that were assessed for self-declared physical activity, Seabra et al. found lower correlations for parent-offspring pairs of opposite genders (r = 0.05 for father-daughter and r = 0.12 for mother-son) when compared to parent-offspring pairs of the same gender (r = 0.12 for father-son and r = 0.18 mother-daughter) [26].

Qualitative studies provide some insight into the mechanisms that could explain gender differences in parent-offspring physical activity correlations. A recent study using semi-structured interviews suggested that mothers are more likely than fathers to pair off with children because of complexities of schedules in two-parent households [27]. Consistent with this hypothesis of mother-offspring shared activities of daily living, our data show that mothers from our population work fewer hours than fathers. The situation might be more complex, however, as we found mother-offspring correlations for ambulatory activity that were higher in employed mothers. Mother-offspring correlations for ambulatory activity were also higher in mothers who reported leisure-time physical activity above the median. The reason for these results are unclear and deserves further investigation of the respective influences of role modeling, sharing of activities, or support by active mothers. Job type might also be important to consider. Recent evidence from the NHANES survey showed that women with full-time sedentary jobs have less light and lifestyle intensity activity than nonworkers on weekdays [28]. Job duration may have an additional influence. In the present study, there was an inverse correlation, in employed mothers, between hours worked and leisure-time physical activity (h·week^−1^) (Spearman rho = −0.23, p = 0.012).

Although our study does not have a longitudinal design, the cross-sectional data suggest that mother-offspring correlations may remain stable over the transition from childhood to adolescence. This is an original and unexpected finding because shared activities, role modeling, the support of active parents, together with other environmental factors, are likely to vary from childhood to adolescence and because ambulatory activity decreased in the offspring with increasing age. Whether this is a generalizable finding is unclear as there is no data to which we could compare our results. However, it should be noted that the characteristics of the study population were similar to those described elsewhere: the mean number of daily steps in children and adolescents was similar to that reported in other studies [29], there was a decrease in ambulatory activity from childhood to adolescence similar to that observed by others at the end of primary school (10–11 years of age) described previously as a pivotal period of change [14], [15], and our data are consistent with the well-documented lower activity levels of girls compared to boys [30].

Correlations were generally higher during weekend days compared to weekdays. It is intuitive that family members spend more time together and share more activities during days out of work/school than during working days. Sharing of activities depends on when parents have the opportunity to be present for their offspring's activity, as suggested in previous studies where parents answered a social support questionnaire that was compared to their children's ambulatory activity [31].

Significant low between-spouse correlations were only present during weekend days, and might be related to shared activities such as shopping together. Interestingly, this observation of almost no significant between-spouse correlation with pedometry is not entirely consistent with previous studies reporting significant between-spouse correlation coefficients for self-reported exercise behavior ranging from 0.16–0.60 [32], [33]. This heterogeneity in results could reflect cohort or cultural differences, or information bias from the use of self-declared data.

Our results improve generalisability for the finding that physical activity correlates within families. Five previous studies used accelerometers derived measures of physical activity in smaller selected samples of families to establish parent-offspring relationships for physical activity. Based on analyses of categorical data, Freedson et al. used Chi^2^ tests to show that familial resemblance occurred in 67% (father-child) and 73% (mother-child) of the families of children 5–9 years [8]. Moore et al. showed that the relative odds ratios of being active for the children of active mothers, or active fathers, or both active parents of children 4–7 years were 2.0, 3.5, and 5.8, respectively [9]. More recently, Oliver et al. showed in children aged 2–5 years that parental physical activity was related to that of their children in multivariate analysis [11]. Fuemmeler et al. showed that parents' physical activity was positively correlated with that of their children (mean age 9.9 years) [12]. Only Jago et al. failed to find association between physical activity of parents and that of their 10–11 years children although they were able to show associations for sedentary time [10].

It is interesting to compare the magnitude of the correlation coefficients we observed with ambulatory activity to those of familial correlation studies that were conducted either on overall physical activity or other behavioral or physiological traits. In 375 nuclear families living in the Quebec City area, Pérusse et al. found ICCs of similar magnitude compared to our data (0.16 for parent-offspring pairs and 0.42 for sibling pairs) for self-reported habitual physical activity [33]. In general, the correlations observed with our pedometer data appear to be of a similar magnitude when compared to other familial outcomes or traits, either behavioral or physiological. Behavioral outcomes such as energy intake have been found to aggregate in families with weak to moderate parent-child correlations (r = 0.20–0.33) [34], [35]. Physiological outcomes such as blood pressure levels [36], weight, height, and BMI (r = 0.29–0.44 between parents and daughters) [37], and muscular strength and endurance (0.14≤r≤0.55 for parent-offspring and sibling correlations) [32] also display significant familial aggregation.

### Limitations

Our study has limitations. First, pedometers do not allow to assess ambulatory behavior in a comprensive way. Pedometers cannot measure duration, frequency or intensity of physical activity (i.e. discriminate between steps accumulated in walking or running for instance) [38]. Second, family data are used to document phenotype similarities among family members, such as those shown in parent–offspring correlations and sibling correlations. These correlations, however, reflect a mix of cultural and genetic transmission. The relations found herein may therefore vary strongly in different populations and settings. Third, our dataset did not allow for an analysis of the effect of the number of children in the family, and especially of the number of younger children, as a putative modifier on the observed correlations.

In conclusion, our data give support to the idea that pedometer-assessed ambulatory activity aggregates within families, with a possible mother effect. The data also showed that mother-offspring correlations remained significant through the transition from childhood to adolescence. Further studies are required to better understand the respective influences of shared activities and parental modeling and support on the familial aggregation of physical activity. However, walking-based activities account for a major portion of physical activity energy expenditure and have been shown to confer substantial health benefits [2]. These data may therefore help to design intervention strategies for the promotion of habitual physical activity at community and family levels.
